# Discovery of molecular determinants of response to targeted therapies in colorectal cancer using patient-derived xenografts (‘xenopatients’)

**DOI:** 10.1186/1753-6561-7-S2-P26

**Published:** 2013-04-04

**Authors:** Francesco Sassi, Giorgia Migliardi, Eugenia Zanella, Francesco Galimi, Francesca Cottino, Andrea Bertotti, Livio Trusolino

**Affiliations:** 1 Laboratory of Molecular Pharmacology, Institute for Cancer Research and Treatment (IRCC), 10060 Candiolo (Torino), Italy; 2 Department of Oncological Sciences, University of Torino Medical School, Institute for Cancer Research and Treatment (IRCC), 10060 Candiolo (Torino), Italy

## Background

Only a fraction of patients with metastatic colorectal cancer (mCRC) receive clinical benefit from therapy with anti-epidermal growth factor receptor (EGFR) antibodies. Gene mutations along the Ras pathway (KRAS, NRAS, BRAF, PIK3CA) correlate with poor response to anti-EGFR antibodies.

## Patients and methods

By implantation and serial propagation in NOD/SCID mice, we produced large xenograft cohorts from 85 patient-derived mCRC samples ("xenopatients").

## Results

Xenopatients retained the histological and genomic features of the original counterparts, responded to the anti-EGFR antibody cetuximab similarly to clinical observations, and could be prospectively stratified as responders or nonresponders based on predictive biomarkers. Genotype-response correlations indicated HER2 amplification specifically in a subset of cetuximab-resistant, KRAS/NRAS/BRAF/PIK3CA wild-type cases. In this subset, combined HER2/EGFR inhibition induced long-lasting tumor regression. We also assessed the effects of MEK and PI3K/mTor inhibitors (AZD6244 and BEZ235 respectively) in 40 specimens harboring KRAS/ NRAS/BRAF/PIK3CA mutations. Cotreatment of xenografts with AZD6244+BEZ235 induced disease stabilization in the majority of cases (70%) but did not lead to tumor regression. Monotherapy was less effective, with BEZ235 displaying higher activity than AZD6244. Triple therapy with cetuximab provided further advantage. The extent of disease control declined upon prolonged treatment.

## Conclusions

Our preclinical platform prospectively recapitulated biomarker-based case stratification and was instrumental in identifying HER2 as a predictor of resistance to cetuximab and of response to combination therapies against HER2 and EGFR. The prevalent growth-suppressive effects produced by MEK and PI3K/mTOR inhibition suggest that this strategy may retard disease progression in KRAS-mutant mCRC patients.

## Financial support

AIRC, Associazione Italiana per la Ricerca sul Cancro; FPRC, Fondazione Piemontese per la Ricerca sul Cancro.

